# Novel Use of Surveillance Data to Detect HIV-Infected Persons with Sustained High Viral Load and Durable Virologic Suppression in New York City

**DOI:** 10.1371/journal.pone.0029679

**Published:** 2012-01-24

**Authors:** Arpi S. Terzian, Sara D. Bodach, Ellen W. Wiewel, Kent Sepkowitz, Marie-Antoinette Bernard, Sarah L. Braunstein, Colin W. Shepard

**Affiliations:** 1 New York City Department of Health and Mental Hygiene, New York, New York, United States of America; 2 Memorial Sloan-Kettering Cancer Center, New York, New York, United States of America; University of Montreal, Canada

## Abstract

**Background:**

Monitoring of the uptake and efficacy of ART in a population often relies on cross-sectional data, providing limited information that could be used to design specific targeted intervention programs. Using repeated measures of viral load (VL) surveillance data, we aimed to estimate and characterize the proportion of persons living with HIV/AIDS (PLWHA) in New York City (NYC) with sustained high VL (SHVL) and durably suppressed VL (DSVL).

**Methods/Principal Findings:**

Retrospective cohort study of all persons reported to the NYC HIV Surveillance Registry who were alive and ≥12 years old by the end of 2005 and who had ≥2 VL tests in 2006 and 2007. SHVL and DSVL were defined as PLWHA with 2 consecutive VLs ≥100,000 copies/mL and PLWHA with all VLs ≤400 copies/mL, respectively. Logistic regression models using generalized estimating equations were used to model the association between SHVL and covariates. There were 56,836 PLWHA, of whom 7% had SHVL and 38% had DSVL. Compared to those without SHVL, persons with SHVL were more likely to be younger, black and have injection drug use (IDU) risk. PLWHA with SHVL were more likely to die by 2007 and be younger by nearly ten years, on average.

**Conclusions/Significance:**

Nearly 60% of PLWHA in 2005 had multiple VLs, of whom almost 40% had DSVL, suggesting successful ART uptake. A small proportion had SHVL, representing groups known to have suboptimal engagement in care. This group should be targeted for additional outreach to reduce morbidity and secondary transmission. Measures based on longitudinal analyses of surveillance data in conjunction with cross-sectional measures such as community viral load represent more precise and powerful tools for monitoring ART effectiveness and potential impact on disease transmission than cross-sectional measures alone.

## Introduction

Surveillance data with expanded HIV-related lab reporting can be used to monitor the HIV epidemic and inform community-wide HIV prevention, care, and treatment efforts. High HIV viral load (VL) among HIV-infected persons is associated with clinical progression to AIDS and death and increased risk for secondary transmission to sex- and needle- sharing partners.[1], [2], [3], [4] Durable virologic suppression, achieved by initiation of highly active antiretroviral therapy (HAART) and engagement in regular HIV-medical care, is necessary to restore immune status, increase survival, and reduce risk of transmission to HIV-uninfected partners. [5], [6], [7], [8] Until recently, surveillance systems did not collect data related to the care and treatment of HIV/AIDS. HIV-related laboratory results like VL, CD4 counts, and genotype testing, now reportable to public health departments in many areas of the US, serve as surrogate markers of linkage to and retention in HIV-related medical care.[9], [10] Such longitudinal data are a source of the type of population-based indicators that have long been desired by HIV policymakers.[11] Furthermore, the Centers of Disease Control (CDC)-led community trials underway in Washington, DC, the New York City borough of the Bronx, Chicago, Houston, Miami, and Philadelphia, use HIV surveillance data to evaluate the feasibility of an enhanced test-and-treat strategy (“TLC-Plus”), which aims to prevent new HIV infections through expanding treatment coverage, improving HIV testing rates and linkage to and retention in HIV care.[12] TLC-Plus and other community-level initiatives rely on HIV-related laboratory reporting to measure and reduce community viral load, an action recommended by the National HIV/AIDS Strategy (NHAS) as part of its larger goal to eliminate HIV-related health disparities.[13]

Recently-published analyses of VL data reported to municipal HIV surveillance systems values have utilized a novel, ecologic measure, community viral load (CVL), as an efficient means of summarizing treatment effectiveness and assessing the success of test-and-treat strategies in a population. In analyses in San Francisco and British Columbia, lower CVL has been shown to correlate with decreased HIV incidence and fewer new HIV diagnoses.[14], [15] While CVL is an appropriate summary measure that provides an overall snapshot of a dynamic cohort, the measure is based on a serial cross-sectional design that limits the ability to make causal inferences on transmission because viral load results cannot be linked back to a given individual.[14] A high CVL may not equate to high VL burden in a particular group as VLs can vary depending on treatment status and risk behaviors at the individual and group-level. Estimates based on repeated VL measures over time may better identify groups with high VL burden as they represent persons with sustained high viremia. However, estimates from both serial cross-sectional and longitudinal analyses are complementary. CVL has the potential to identify high-risk groups that may need additional prevention efforts, while longitudinal estimates help decipher what underlies the CVL, summarizing the dynamics of control of VL at the individual and group level. Taken together, these measures may help target HIV care and treatment interventions to the subpopulations whose viral control – if achieved - will most likely result in rapid lowering of CVL.

The current analysis extends CVL analyses by using longitudinal data to define novel summary measures in an HIV-infected population: Sustained high viral load (SHVL), and durably-suppressed viral load (DSVL). We used these measures to evaluate community-wide control of HIV viremia in NYC in 2006–2007, and to identify factors associated with SHVL, with the hypothesis that SHVL is more common among groups known to have suboptimal engagement in HIV-related medical care.

## Methods

### Ethics Statement

Data were collected as part of legally-mandated public health surveillance for HIV in New York State, according to Chapter 163 of New York State Public Health Law, Article 21, Title 3. Analysis of HIV surveillance data is authorized for the purpose of epidemiologic monitoring, as specified in the New York Codes, Rules and Regulations, Title 10, Part 63 (Revised 6/2005). This analysis, using existing and de-identified data, qualifies as non-research according to the Department of Health and Human Services (DHHS) regulations for the protection of human rights in 45 CFR §46.102[d]. For these reasons, this analysis is exempt from human subjects review requirements.

### Data Sources

The NYC HIV Surveillance Registry (Registry) is a population-based registry of all persons diagnosed with HIV infection or AIDS, as defined by the CDC, and reported to the NYC DOHMH.[16] Name-based reporting of AIDS diagnoses was mandated by New York State (NYS) law in 1983, followed by HIV reporting in 2000 and laboratory reporting of all positive Western Blots, viral loads, CD4 counts, and nucleotide sequence results in 2005.[17] All incoming electronic laboratory reports are matched to cases in the Registry.[10] Vital status for all cases in the Registry is updated through quarterly matches to the NYC Death Registry and periodic matches to national death data. Data for this analysis were reported to the NYC DOHMH as of December 31, 2009.

### Eligibility criteria

All persons diagnosed with HIV infection reported to the NYC DOHMH by December 31, 2009 and presumed to be living with HIV/AIDS as of the end of December 2005 were eligible for inclusion. Cases that were at least 12 years of age by December 31, 2005, had a NYC residence at HIV or first-AIDS diagnosis, and had two or more HIV RNA viral load tests reported between January 1, 2006 and December 31, 2007 were included in the analysis. VLs reported less than two weeks apart for the same individual were censored to maximize the possibility that VL values represented a deliberate attempt to monitor clinical status with serial measurements rather than tests repeated for quality control or other reasons.

### Analytic Variables

#### Outcome variables

HIV VL results were quantified using VL testing kits that were commercially-available and in use in NYC at the time. Results were reported as a continuous value or as the limit of detection of the testing kit used. Three analytic and mutually exclusive variables were constructed for each individual: Peak VL, SHVL, and DSVL. For descriptive analyses, peak VL was defined as the maximum VL value observed for an individual between January 1, 2006 and December 31, 2007. Peak VLs were categorized into three groups: 1) Undetectable: 0 to ≤400 copies/mL; 2) Other: 401 to 99,999 copies/mL); and 3) High: ≥100,000 copies/mL. For descriptive analyses, SHVL was defined as an individual ever having a pair of consecutive VLs ≥100,000 copies/mL. To measure the duration of SHVL, we defined SHVL in statistical models as a proportion, the number of consecutive VL pairs ≥100,000 copies/mL out of total pairs observed. An interval was defined as the difference in time between the first and second test dates within a pair of consecutive VLs.

#### Independent variables

Analytic variables included HIV transmission risk, age, race/ethnicity, sex, borough of residence, history of an AIDS diagnosis, and presence of concurrent AIDS at the time of HIV diagnosis. We applied the CDC hierarchy to create transmission risk categories to guide risk assignment, especially for cases with more than one reported risk.[18] Transmission risk categories included injection drug use (IDU), men who have sex with men (MSM), heterosexual sex, and other. Heterosexual risk included persons who reported having heterosexual sex with an HIV-infected person, an IDU, or a person who had received blood products. For females only, heterosexual risk also included sex with a male and at least one of the following: history of commercial sex work, multiple male sex partners, sexually transmitted disease, crack/cocaine, sex with a bisexual male, probable heterosexual transmission as noted in a medical chart, or negative history of IDU. Other risk included persons with perinatal, transfusion, transplant, and unknown risk.

#### Covariates

Age was defined as the difference in years between date of birth and December 31, 2005. Race/ethnicity categories included non-Hispanic black, Hispanic, non-Hispanic white and ‘other.’ Persons of Hispanic ethnicity, regardless of race, were classified as Hispanic. Due to small numbers, Asian/Pacific Islanders, Native Americans, and persons reporting multiple, other or unknown race were grouped as ‘Other.’ Borough, which designates the five county geography of NYC, referred to the borough of residence at the time of HIV or AIDS diagnosis. Time since diagnosis was treated as a binary variable, with a cutoff at 5 years, representing the 25^th^ percentile of infection times, defined as the difference from date of diagnosis to December 31, 2005.

### Data Analysis

The population analyzed was compared to all PLWHA in the Registry as of December 31, 2005 to assess whether there were differences by sociodemographic characteristics. Within the population analyzed, comparisons were made between individuals with SHVL and DSVL. All comparisons were tested using χ^2^ tests of association. Marginal mean models were specified, relating the response variable, SHVL, to covariates and the working correlation structure between the probabilities of SHVL at different time points, using generalized estimating equations (GEE) methods.[19] In multivariate models, estimates were adjusted for age, race/ethnicity, sex, borough of residence, concurrent HIV/AIDS status, and time since diagnosis. SAS v9.2 software was used to conduct the analysis (SAS Institute Inc., Cary, NC).

## Results

**Figure 1:**
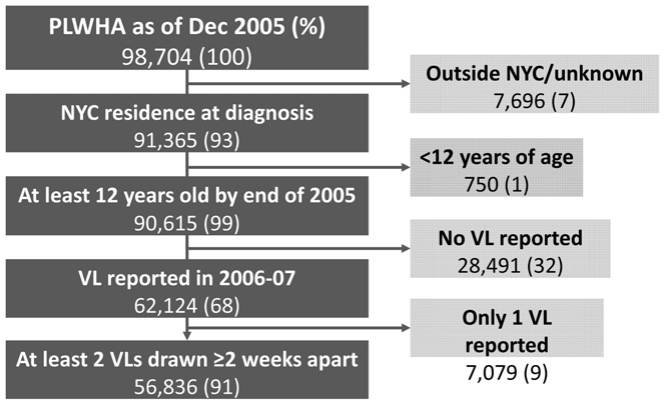
Eligibility flow chart on HIV-infected New Yorkers, 2006 and 2007.

**Figure 2:**
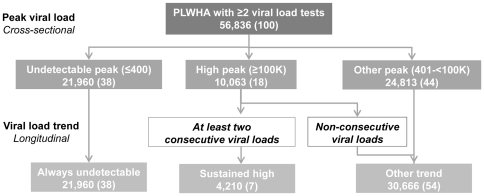
HIV-infected New Yorkers grouped by VL trend, 2006–2007.

The population analyzed included 56,836 PLWHA, representing nearly 60% of all PLWHA diagnosed and presumed living in NYC at the end of 2005 (Figure 1). PLWHA with ≥2 VLs were demographically similar to all PLWHA at the end of 2005 (Table 1). Two-thirds of PLWHA with ≥2 VLs were male and nearly one-third had a reported MSM risk. Nearly one-quarter had IDU risk. The proportion concurrently diagnosed with HIV and AIDS was 24%. Nearly two-thirds had an AIDS diagnosis by the end of 2005. Almost 40% had DSVL, meaning all VLs were ≤400 copies/mL. Fifteen percent had a peak VL ≥100,000 copies/mL (N = 8,403). Among those with a high peak VL, 52% had a VL ≥100,000 only once in the two years and 40% (N = 4,210) had SHVL, meaning at least two consecutive VLs ≥100,000 (Figure 2). Compared to all PLWHA, those with SHVL were slightly younger (median age 42 vs.44 years), more likely to be non-Hispanic black (50% vs. 45%), female (36% vs. 30%), and have a higher median number of tests (7 vs. 5; Table 2). They were also more likely to have died by the end of 2007 (11% vs. 3%; Table 2) and at a younger age, nearly 10 years younger, than those without SHVL (data not shown). Among those who died, the proportion with IDU risk in the SHVL and DSVL groups was 35% and 45%, respectively.

**Table 1:** Characteristics of HIV-infected New Yorkers with ≥2 viral load tests and all persons diagnosed and presumed living, New York City^1^.

Characteristic	Subcategory	PLWHAwith ≥2 testsN (%)	All PLWHAas of 12/2005N (%)
**Overall**		**56,836**	**98,704**
**Risk** 2			
	MSM	17,076 (30)	29,559 (30)
	IDU	12,624 (22)	22,703 (23)
	Other/Unknown	15,520 (27)	28,422 (29)
	Heterosexual	11,616 (20)	17,887 (18)
**Age in years**			
	Mean (Med)	44 (44)	44 (44)
**Sex**			
	Female	18,661 (33)	29,920 (30)
	Male	38,175 (67)	68,651 (70)
**Race/ethnicity** 3			
	Non-Hispanic Black	26,214 (46)	44,310 (45)
	Non-Hispanic White	11,121 (20)	20,543 (21)
	Hispanic	19,177 (34)	31,812 (33)
	Other	324 (<1)	802 (<1)
**Borough of residence** 4			
	Bronx	14,105 (25)	21, 409 (22)
	Brooklyn	15,291 (27)	24,376 (25)
	Manhattan	18,638 (33)	29,807 (30)
	Queens	7,752 (14)	13,596 (14)
	Staten Island	1,050 (2)	1,780 (<2)
	Outside of NYC	0	7,589 (8)
	Unknown/Missing	0	14 (<1)
**Concurrent HIV/AIDS diagnosis**			
	Yes	13,359 (24)	25,246 (26)
	No	43,477 (76)	73,458 (74)
**Time since diagnosis in years**			
	Mean (Median)	8 (7)	8 (7)
**AIDS as of 2005**			
	N (%)	21,978 (62)	59,486 (60)
	Time since AIDS in years		
	Mean (Median)	6 (6)	7 (7)
**VL tests from 2006 to 2007**			
	Mean (Median)	6 (6)	5 (5)

VL, viral load. NYC, New York City. PLWHA, persons living with HIV/AIDS. MSM, men who have sex with men. IDU, intravenous drug users.

1 Data as reported to the NYC DOHMH by December 31, 2009.

2 Includes persons who had heterosexual sex with an HIV-infected person, an injection drug user, or a person who has received blood products. For females only, heterosexual sex also includes sex with a male and at least one of the following: history of commercial sex work, multiple male sex partners, sexually transmitted disease, crack/cocaine use, sex with a bisexual male, probable heterosexual transmission as noted in a medical chart, or negative history of injection drug use.

3 NYC DOHMH collects race and ethnicity data that meet federal standards of classification and maintains ethnicity data separately from race information. Persons of Hispanic or Latino ethnicity have a separate race classification. Due to small numbers, persons reporting more than one race, Native Americans or Alaska Natives, Hawaiian Natives, Asian, and Pacific Islanders were classified as ‘Other’.

4 Borough of residence refers to the residence at HIV diagnosis for persons living with HIV (non-AIDS) or residence at AIDS diagnosis for PLWHA.

**Table 2:** Characteristics of HIV-infected New Yorkers with sustained high viral load (SHVL) and durable virologic suppression and all persons diagnosed and presumed living, New York City^1^.

Characteristic	Subcategory	Sustained high viral loadN (%)	Durably suppressedN (%)	All PLWHAas of 12/05N (%)
**Overall**		**4,210**	**21,960**	**98,704**
**Risk** 2				
	MSM	1,058 (25)	7,592 (35)	29,556 (30)
	IDU	1,113 (26)	4,104 (19)	22,616 (23)
	Other/Unknown	1,144 (27)	5,981 (27)	26,297 (29)
	Heterosexual	895 (21)	4,283 (20)	17,780 (18)
**Age in years**				
	Mean (Med)	41 (42)	47(46)	44 (44)
	IQR	36, 47	40, 53	38, 51
**Sex**				
	Female	1,502 (36)	6,562 (30)	29,973 (30)
**Race/ethnicity** 3				
	Non-Hispanic Black	2,141 (50)	8,998 (40)	44,478 (45)
	Non-Hispanic White	585 (14)	5,492 (25)	20,617 (21)
	Hispanic	1,473 (35)	7,321(33)	31,715 (32)
	Other	11 (<2)	149 (2)	1,894 (<2)
**Borough of residence** 4				
	Bronx	1,202 (29)	4,667 (21)	21, 409 (22)
	Brooklyn	1,196 (28)	5,517 (25)	24,376 (25)
	Manhattan	1,247 (30)	7,981 (36)	29,807 (30)
	Queens	488 (11)	3,402 (16)	13,596 (14)
	Staten Island	77 (2)	3jk93 (2)	1,780 (<2)
	Outside of NYC	0	0	7,589 (8)
	Unknown/Missing	0	0	14 (<1)
**Concurrent HIV/AIDS at diagnosis**				
	Yes	1,021 (24)	6,260 (29)	26,279 (27)
	No	3,189 (76)	15,700 (71)	72,427 (74)
**Time since diagnosis in years**				
	Mean (Median)	9 (8)	9 (8)	8 (7)
	IQR	5, 13	5, 12	4, 12
**AIDS as of 12/05**				
	N (%)	3,123 (74)	14,236 (65)	59,492 (60)
	Time since AIDS in years			
	Mean (Median)	6 (6)	7 (7)	7 (7)
**Deaths as of 12/07**				
	N (%)	481 (11)	464 (2)	2,545 (3)
**VL tests, 2006–2007**				
	Mean (Median)	7 (7)	6 (6)	5 (5)

VL, viral load. NYC, New York City. PLWHA, persons living with HIV/AIDS. MSM, men who have sex with men. IDU, intravenous drug use. IQR, interquartile range. NA, not available.

1 Data as reported to the NYC DOHMH by December 31, 2009.

2 Includes persons who had heterosexual sex with an HIV-infected person, an injection drug user, or a person who has received blood products. For females only, heterosexual sex also includes sex with a male and at least one of the following: history of commercial sex work, multiple male sex partners, sexually transmitted disease, crack/cocaine use, sex with a bisexual male, probable heterosexual transmission as noted in a medical chart, or negative history of injection drug use.

3 NYC DOHMH collects race and ethnicity data that meet federal standards of classification and maintains ethnicity data separately from race information. Persons of Hispanic or Latino ethnicity have a separate race classification. Due to small numbers, persons reporting more than one race, Native Americans or Alaska Natives, Hawaiian Natives, Asian, and Pacific Islanders were classified as ‘Other’.

4 Borough of residence refers to the residence at HIV diagnosis for persons living with HIV (non-AIDS) or residence at AIDS diagnosis for PLWHA.

Table 3 summarizes estimates from univariate and multivariate GEE regression models. IDU risk remained significantly associated with SHVL compared to heterosexual risk, after adjusting for age, sex, race, borough of residence, concurrent HIV/AIDS status, and time since diagnosis (adjusted odds ratio [AOR] = 1.34; 95% Confidence Interval [CI] = 1.19,1.50). MSM had significantly decreased risk for SHVL compared with heterosexuals. Compared with persons aged 50+, all younger age groups were at increased risk for SHVL, though the youngest group were at highest risk (AOR = 3.42; 95% CI = 2.72, 4.32).

**Table 3:** Univariate and multivariate GEE regression models identifying factors associated with sustained high viral load in 2006–2007, New York City (N = 56,836)^1^.

Characteristic	Subcategory	PLWHA with ≥2 testsN (%)	Crude OR(95% CI)^2^	Adjusted OR(95% CI)^2,3^
**Risk** 4				
	MSM	17,076 (30)	0.74 (0.66, 0.82)	0.80 (0.70, 0.91)
	IDU	12,624 (22)	1.16 (1.04, 1.29)	1.34 (1.19, 1.50)
	Other	15,520 (27)	1.02 (0.92, 1.13)	1.00 (0.90, 1.12)
	Heterosexual	11,616 (20)	1.00	1.00
**Age**				
	13–19	1,038 (<2)	3.56 (2.85, 4.46)	3.42 (2.72, 4.32)
	20–29	3,438 (6)	2.48 (2.12, 2.89)	3.09 (2.63, 3.63)
	30–39	12,175 (21)	2.48 (2.22, 2.70)	2.86 (2.56, 3.19)
	40–49	23,240 (41)	1.92 (1.74, 2.12)	2.02 (1.80, 2.24)
	≥50	16,945 (30)	1.00	1.00
**Sex**				
	Female	18,661 (33)	1.20 (1.12, 1.30)	0.95 (0.87, 1.04)
	Male	38,175 (67)	1.00	1.00
**Race/ethnicity** 5				
	Non-Hispanic Black	26,214 (46)	1.78 (1.60, 2.00)	1.46 (1.30, 1.65)
	Hispanic	19,177 (34)	1.67 (0.48, 1.88)	1.29 (1.14, 1.46)
	Other	324 (<1)	0.70 (0.36, 1.36)	0.71 (0.37,1.37)
	Non-Hispanic White	11,121 (20)	1.00	1.00
**Borough of residence** 6				
	Bronx	14,105 (25)	1.41 (1.28, 1.55)	1.13 (1.02, 1.25)
	Brooklyn	15,291 (27)	1.24 (1.13,1.36)	1.04 (0.94, 1.14)
	Queens	7,752 (14)	0.98 (0.86, 1.11)	0.87 (0.76, 0.98)
	Staten Island	1,050 (2)	1.21 (0.92, 1.59)	1.15 (0.88, 1.50)
	Manhattan	18,638 (33)	1.00	1.00
**Concurrent HIV/AIDS at diagnosis**				
	Yes	13,359 (24)	1.05 (0.96, 1.14)	1.11 (1.02, 1.21)
	No	43,477 (76)	1.00	1.00
**Time since diagnosis in years**				
	0–4	17,225 (30)	1.04 (0.96, 1.12)	0.93 (0.85, 1.01)
	≥5	39,661 (70)	1.00	1.00

GEE, generalized estimating equations. VL, viral load. MSM, men who have sex with men. IDU, intravenous drug users.

1 Data as reported to the NYC DOHMH by December 31, 2009.

2 Adjusted for number of VL pairs observed, 2006–2007.

3 Adjusted for age, race/ethnicity, borough, sex, history of AIDS, concurrent HIV/AIDS status at diagnosis, and number of pairs of VL tests.

4 Heterosexual risk includes persons who had heterosexual sex with an HIV-infected person, an injection drug user, or a person who has received blood products. For females only, heterosexual sex also includes sex with a male and at least one of the following: history of commercial sex work, multiple male sex partners, sexually transmitted disease, crack/cocaine use, sex with a bisexual male, probable heterosexual transmission as noted in a medical chart, or negative history of injection drug use.

5 NYC DOHMH collects race and ethnicity data that meet federal standards of classification and maintains ethnicity data separately from race information. Persons of Hispanic or Latino ethnicity have a separate race classification. Due to small numbers, persons reporting more than one race, Native Americans or Alaska Natives, Hawaiian Natives, Asian, and Pacific Islanders were classified as ‘Other.’

6 Borough of residence refers to the residence at HIV diagnosis for persons living with HIV (non-AIDS) or residence at AIDS diagnosis for persons living with AIDS.

Non-Hispanic blacks and Hispanics were 46% and 29% more likely to have SHVL than whites, respectively (AOR = 1.46; 95% CI = 1.30,1.65; AOR = 1.29; 95% CI = 1.14, 1.46). PLWHA with concurrent HIV/AIDS at diagnosis were 11% more likely than those without concurrent diagnosis to have SHVL (AOR = 1.11; 95% CI = 1.02, 1.21). Bronx residents were 13% more likely to have SHVL than Manhattan residents (AOR = 1.13; 95% CI = 1.02, 1.25). No significant associations were observed between SHVL and sex or time since diagnosis.

## Discussion

We used public health HIV surveillance data to create novel summary measures that track HIV VL dynamics at the population level, and applied these longitudinal measures to the known population of HIV-infected New Yorkers receiving HIV-related medical care in NYC in 2006 and 2007. Findings show that analytic population had an average of six VL test results, suggesting frequent engagement in HIV-related medical care. Almost 40% had virologic suppression for two years, indicating sustained treatment success.

Previous studies have shown that IDUs and persons of color were more likely to delay initiation and have inconsistent utilization of HIV-related care compared to non IDUs and non-Hispanic whites in NYC.[9], [10] Findings that show IDUs, blacks, and Hispanics at increased risk for SHVL support our hypothesis that SHVL is more common among groups known to have suboptimal engagement in HIV-related medical care. IDUs face challenges in achieving timely linkage to and retention in care.[20]–[25] Delayed initiation of care is associated with poorer immunologic recovery and increased risk of death.[8], [10], [26] Our analysis suggests that IDUs are an appropriate focus for public health programs aimed at increasing engagement in care among groups most at-risk for HIV-related morbidity.

Blacks, accounting for more than half of PLWHA in the US and with HIV diagnosis rates 8 times that of whites, are also more likely to delay initiation of care after HIV diagnosis. [8], [10], [27], [28], [29] Hispanics, accounting for 17% of all new HIV infections in 2006 and with HIV diagnosis rates 3 times that of whites, have similar barriers to engagement in care. [30], [31] This trend is consistent with the NYC epidemic where blacks and Hispanics accounted for 50% and 33% of all newly diagnosed HIV cases that were concurrently diagnosed with AIDS in 2009, a marker of late-stage disease and missed opportunities for care.[32]

MSM, disproportionately accounting for prevalent and new diagnoses in NYC, have been shown to have timely initiation and high retention in HIV-related medical care, often serving as the referent group for studies on linkage to care.[9], [10], [33] Because engagement in care dramatically improves the probability of achieving virologic suppression, it is not surprising that MSM were over-represented among those with DSVL and were not at increased risk for SHVL in this analysis. While blacks and Hispanics comprise less than 60% of MSM overall, they made up almost 90% of MSM with SHVL. Future analyses on SHVL exploring the interaction of transmission risk and race/ethnicity and other markers of SES such as proportion living below poverty, are needed to adequately describe the black and Hispanic MSM population at increased risk for SHVL.

Bronx residents were at increased risk for SHVL, a finding consistent with other HIV indicators showing increased HIV-related morbidity and mortality in the Bronx compared to the other boroughs. [34] Bronx residence is likely a surrogate marker for other group membership like race/ethnicity and transmission risk. Known health disparities in the Bronx were part of the impetus for the selection of the Bronx for the DOHMH's community-wide HIV testing scale-up effort, “The Bronx Knows”. [35]

One of the most concerning findings was that PLWHA aged 13–19 years had 3 times greater risk of SHVL than PLWHA 50 years or older. The relative youth of the 13–19 years age group belies the likelihood that most are highly ART-experienced, given that more than 80% of them were infected with HIV through perinatal transmission, and were diagnosed during the era of mono and dual therapy before highly active antiretroviral regimens were available for HIV treatment. The SHVL among these perinatally-infected adolescents may stem from the development of ART resistant viral strains, or from the adherence gaps often observed among adolescents with chronic illnesses. [36], [37] Regardless of its causes, the presence of SHVL among PLWHA who have entered their sexually-active and childbearing years has serious implications for HIV transmission. HIV-infected youth often engage in unprotected sex and do not disclose their HIV status to sex partners. [38], [39] HIV-infected youth are also more likely to have a diagnosis of an acute sexually transmitted infection than HIV-infected adults and, among females, to become pregnant. [40] SHVL concomitant with risky sexual behaviors places youth at higher risk for HIV-related morbidity and transmitting to others.

Our analysis is subject to limitations. Findings are based on persons presumed to be in care because they had VL results reported to the NYC DOHMH. More than one third of nearly 100,000 PLWHA reported by the end of 2005 were excluded because they had only one or no VL result reported in 2006–2007. This group may represent persons who: 1) are receiving HIV care outside of NYC; 2) died out of NYC and their death was not yet reported to the NYC DOHMH; 3) are out of care; or 4) received care but not VL testing. Because the HIV-infected population in the US is highly mobile and PLWH relocation to jurisdictions outside of NYC are not routinely reported to DOHMH, it is difficult to estimate the proportion of NYC PLWHA without a recent viral load who are engaged in care in another reporting jurisdiction. Despite this distinction, no differences by sociodemographic characteristics between PLWHA with 2 or more VLs and all PLWHA were observed (Table 1). [41]

Characterization of the ‘other’ group where peak VL was detectable but never ≥100,000 copies/mL was limited. Given the heterogeneity in infection times and treatment experiences, some PLWHA may have a more difficult-to-treat virus where achieving DSVL is unlikely with any level of adherence, and thus cannot be considered the goal of therapy. Refinement of the characterization of HIV viremia in this group by exploring measures like area under the curve (AUC), a commonly used measure to quantify rate of change in biomarkers over time, may provide a more comprehensive measure. [42], [43]

Our ability to infer the level of engagement in care from the number of cumulative VL tests may be limited. We assumed that 1) those more engaged in care had a higher cumulative number of VL tests and; 2) those with the same cumulative number of tests were similar in their access and utilization of care. However, those with the same number of VL tests may be quite different. Among persons with fewer VL tests, for example, some may be non-adherent, experiencing virologic failure and missing the visits at which their VLs would be drawn, while others may be durably suppressed on a stable ART regimen, and therefore require less frequent clinical monitoring. Lastly, VL tests do not necessarily mean that meaningful HIV care was provided to the person tested by a qualified HIV clinician, or that results were received and informed treatment.[33]

Our analyses show that DSVL and SHVL detected HIV disparities, similar to more established HIV population indicators like HIV incidence, diagnosis, and death rates. [44] Because undetectable VL is associated with reduced transmission and improved health outcomes, achieving suppression, a specific target of the NHAS, necessitates use of longitudinal measures. [45]–[47] Our markers not only complement CVL as basic measures of treatment coverage and effectiveness, but improve measurement by characterizing longitudinal patterns of VL dynamics within a population. Moreover, identifying SHVL provides a specific opportunity for outreach to those HIV-infected individuals in a community who are at greatest risk of near-term HIV-related morbidity and mortality. In 2011, DOHMH began active outreach among persons with SHVL who appeared to lack medical follow-up in the months that followed their very high viral load, with the goal of interviewing these clients about partner services and assisting them with returning to medical care for HIV. Prior to this analysis, such outreach was not specifically directed or prioritized based on the magnitude of HIV VL prior to interruption of care. Future analyses will 1) characterize longer-term trajectories of persons with SHVL and DSVL, using AUC to help distinguish rates of virologic change particularly among those with the same number of tests, 2) characterize genotype profiles among SHVLs and measure the proportion who have resistant strains and 3) investigate the relationship between the number of newly reported HIV diagnoses and number of PLWHA with SHVL and DSVL to evaluate its potential as a surrogate marker of incidence. Altogether, findings support the use of these surveillance markers to evaluate treatment effectiveness and HIV transmission risk, especially in high risk groups.
